# Overcoming Data Loss in Wearable Disease Detection with GAN-Based Imputation

**DOI:** 10.1038/s41746-026-02518-4

**Published:** 2026-03-27

**Authors:** Jürgen Wallner, Sarah Berbuir, Lukas Birner, Adrian Dendorfer, Bivek Panthi, Beatrix Rahnsch, Julius Muma, Stephen Munga, David Obor, Till Bärnighausen, Sandra Barteit

**Affiliations:** 1 Heidelberg Institute of Global Health (HIGH), Faculty of Medicine and University Hospital, Heidelberg University, Heidelberg, Germany; 2 TUM School of Computation, Information and Technology (CIT), Technical University of Munich (TUM), Munich, Germany; 3 Kenya Medical Research Institute (KEMRI), Nairobi, Kenya; 4 Africa Health Research Institute (AHRI), KwaZulu-Natal, South Africa; 5 Harvard Center for Population and Development Studies, Cambridge, MA, USA

**Keywords:** Computational biology and bioinformatics, Diseases, Health care, Mathematics and computing, Medical research

## Abstract

High rates of missing data in wearable sensor streams hinder early detection of infectious diseases, especially in low-resource settings with inconsistent device adherence and connectivity. We developed a lightweight generative adversarial network (GAN) framework that imputes missing heart rate data and integrates with a rule-based anomaly detection algorithm to identify early signs of infection. In a cohort from rural Kenya (*n* = 300, 161 malaria-positives), our system triggered early alerts in 100 cases, including 42 solely with imputation. Alerts preceded symptom onset by 11.9 days, aligning with the 11.7-day parasitemia window from controlled trials. Despite 50% data coverage, alerts occurred on 3.5 consecutive days during the infection window, improving early detection by 35%. The GAN, trained only on external COVID-19 data (*n* = 3318), generalized to malaria, reducing reconstruction error by 58%. This approach demonstrates scalable, cross-pathogen physiological monitoring and offers a robust tool for disease surveillance in settings challenged by high wearable data loss.

## Introduction

Continuous physiological monitoring through wearable sensors enables early detection of infectious diseases and clinical deterioration by capturing subtle changes in vital signs—such as heart rate (HR), temperature, and respiratory rate—before overt symptoms appear. These deviations often reflect autonomic or inflammatory responses to infection, allowing for earlier identification of illness onset compared to symptom-based or episodic clinical assessments^1,2^. Beyond infectious diseases, wearable technologies have also demonstrated utility in monitoring and managing non-communicable conditions such as cardiovascular disease^3^, diabetes^4^, and sleep disorders^5^, supporting broader applications in longitudinal health surveillance.

This capability holds particular promise in decentralized and resource-limited settings, where sparse diagnostic infrastructure and delayed care-seeking often lead to adverse outcomes^6^. In such contexts, access to laboratory diagnostics and clinical personnel is frequently limited^7,8^, and patients may not seek care until symptoms become severe^9^. Wearable sensors offer a scalable means of bridging this gap by providing continuous, passive data collection without requiring facility-based visits, enabling earlier detection and community-level triage that can inform timely interventions and optimize the allocation of scarce healthcare resources.

However, real-world deployments frequently suffer from fragmented data streams due to sensor non-adherence, battery limitations, and unreliable connectivity—issues especially acute in low- and middle-income countries (LMICs). These failures often stem from environmental and socioeconomic constraints, such as limited access to electricity for device recharging, physical discomfort or cultural hesitancy toward continuous wear, and intermittent mobile network coverage. In addition, technical disruptions—including inconsistent device compatibility, firmware instability, and fragmented data pipelines across proprietary platforms—complicate reliable data ingestion, synchronization, and long-term storage. Together, these factors substantially reduce the continuity and utility of wearable-derived data in real-world settings. Additionally, logistical challenges in device maintenance and replacement further compromise data continuity, reducing the reliability of wearable-derived insights in precisely the settings where they are most needed^10^.

Missing data poses critical limitations for several key applications. In randomized controlled trials (RCTs), wearables are increasingly used to capture digital endpoints, such as resting HR or activity levels, as primary or secondary outcomes. Gaps in these data streams can reduce statistical power, introduce bias, and violate assumptions of missing-at-random required for standard analyses. Similarly, in predictive modeling tasks—such as detecting infection onset or physiological deterioration—model performance deteriorates sharply when inputs are sparse or inconsistently sampled, particularly for methods that rely on temporal dependencies. Missingness also undermines applications in long-term disease monitoring, behavioral interventions, and personalized feedback, where consistent input is critical for adaptive guidance and outcome tracking^11^.

At the same time, wearable technologies now enable the collection of continuous, longitudinal physiological data at a population scale—moving beyond the cross-sectional snapshots typical of traditional epidemiological studies. This high-resolution temporal data vastly increases the number of observations per individual, creating new opportunities for early warning, pattern recognition, and adaptive modeling^12–14^. If leveraged effectively, these dense real-world data streams could help establish a foundation for precision public health by enabling timely, individualized interventions and more responsive population-level surveillance^15^. Realizing this potential, however, depends critically on solving the pervasive problem of data fragmentation and loss.

**Fig. 1:**
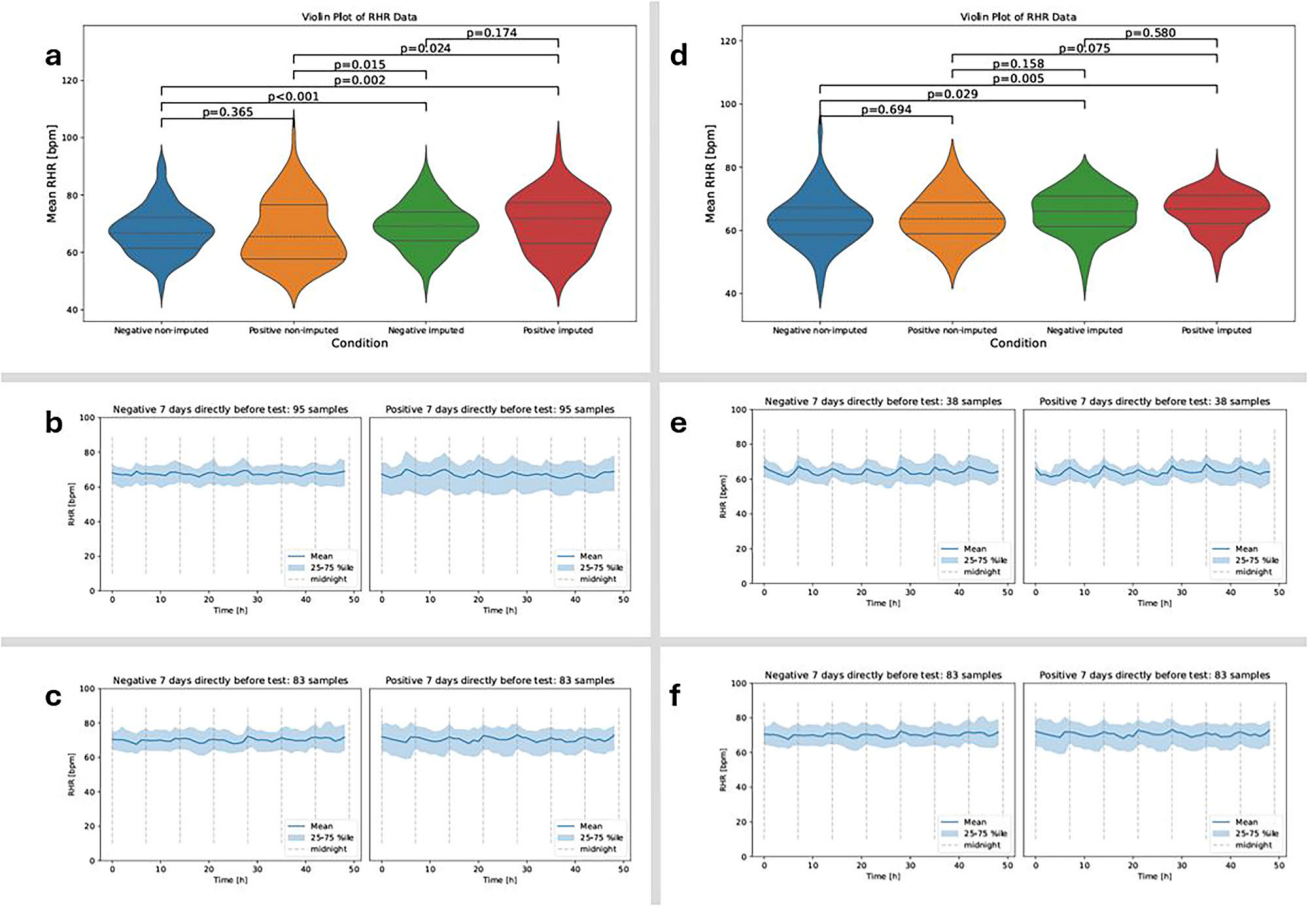
Resting heart rate (RHR) profiles reveal infection-related differences and benefit from GAN-based imputation. **a** COVID-19 cohort: mean RHR distributions for negative (blue) and positive (orange) cases, shown for raw (non-imputed) and GAN-imputed data. **d** Malaria cohort, same layout. In both cohorts, imputation enhances temporal continuity, but while the COVID-19 cohort retains the same overall trend after imputation, the malaria cohort shows a more distinctive separation between health states only after imputation. **b**, **c** Exemplary 7-day segments before a positive COVID-19 test: raw data (**b**) already demonstrate a distinction between positive and negative cases, and imputed data (**c**) simply preserve this pre-diagnostic RHR elevation pattern—expected given the relatively complete recordings in this cohort. **e**, **f** Matched malaria examples, where raw data show little distinction between positive and negative cases due to substantial missingness (**e**); imputation recovers and clarifies these differences (**f**). These results support the hypothesis that wearable data dropout can hide latent physiological signals recoverable through imputation.

Many existing anomaly detection algorithms rely on the availability of high-resolution, regularly sampled data^16^, which is often not feasible in real-world deployments, thereby limiting their reliability and clinical applicability. To address this gap, we developed an interpretable framework that combines a generative adversarial network (GAN)-based imputation model with a rule-based anomaly detection algorithm^2,17^. The GAN reconstructs missing segments of HR time series while preserving physiologically plausible rhythms (Fig. 1) and generalizes across disease contexts without the need for retraining on the target malaria cohort^18^. Trained exclusively on a COVID-19 dataset, it restored detection performance in a separate malaria cohort, highlighting its potential for robust, cross-pathogen surveillance. By mitigating the effects of data loss, this framework enables earlier detection of physiological deviations and may enhance community-level triage and clinical responsiveness in low-resource environments.

**Fig. 2:**
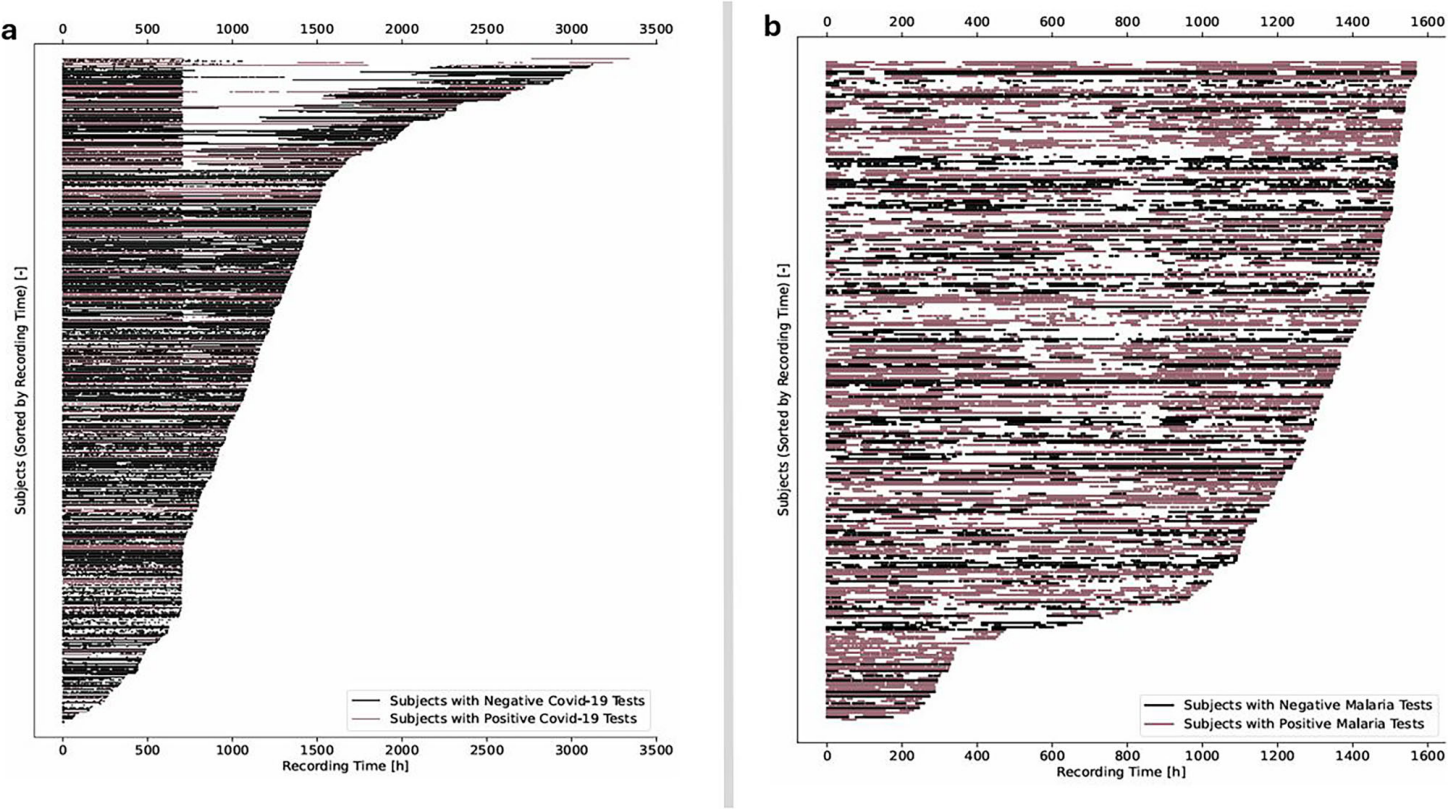
Real-world wearable datasets show distinct data completeness profiles across infectious disease studies. **a** In the COVID-19 cohort, subjects with diagnostic results (black = negative, dark red = positive) generally have longer and more continuous heart rate (HR) recordings, with relatively few short gaps. **b** In contrast, the malaria cohort exhibits shorter overall recordings, more frequent and prolonged signal dropouts, and denser clusters of positive tests. These patterns highlight variability in data quality across study contexts and the importance of imputation strategies that can adapt to different sparsity profiles.

We validated our approach using two longitudinal datasets from wearable devices (Fig. 2). In a prospective study conducted in rural western Kenya (*n* = 300), we detected 100 malaria infection episodes—42 of which would have remained undetected without imputation—with algorithmic alerts occurring an average of 11.9 days before symptom onset. This lead time aligns with known parasitemia dynamics and supports the potential for earlier diagnostic referral. A complementary evaluation in a U.S.-based COVID-19 cohort (*n* = 3318) demonstrated robust physiological signal reconstruction and successfully triggered early alerts for PCR-confirmed infections, confirming cross-pathogen applicability. Together, these findings indicate that our approach can make wearable-based disease detection feasible even in real-world conditions where data are often incomplete. By recovering missing information and identifying early signs of infection, the system can support health workers in low-resource settings by guiding whom to test, when to intervene, and how to prioritize care. This has important implications for public health policy, as it provides a scalable, low-cost tool to strengthen community-based surveillance systems, particularly in areas where laboratory testing is limited or delayed. Such tools could help shift infectious disease detection from reactive to proactive, enabling earlier diagnosis and better allocation of scarce healthcare resources.

## Results

### Fidelity of GAN-based imputation

To evaluate the effectiveness of our GAN-based imputation framework for wearable heart rate (HR) data in infection monitoring, we compared its performance against several standard imputation methods: mean substitution, last observation carried forward (LOCF)^19^, Gaussian process regression (GPR)^20^, and Bayesian Gaussian mixture models (BGMM)^21,22^. Mean substitution and LOCF are simple heuristics that fill missing values using averages or the most recent observation, respectively. In contrast, GPR and BGMM are probabilistic models widely used in biomedical time series analysis. GPR interpolates data using a non-parametric Bayesian framework that captures temporal dependencies, while BGMM models data distributions as mixtures of Gaussian components, enabling uncertainty-aware imputation. These methods were selected to represent a spectrum of commonly used approaches, from simple rules to flexible generative models. Across both the COVID-19 and malaria cohorts, the GAN consistently yielded the lowest mean squared error (MSE) in masked regions, with performance gains most evident in sequences where missing data exceeded 20%.

**Fig. 3:**
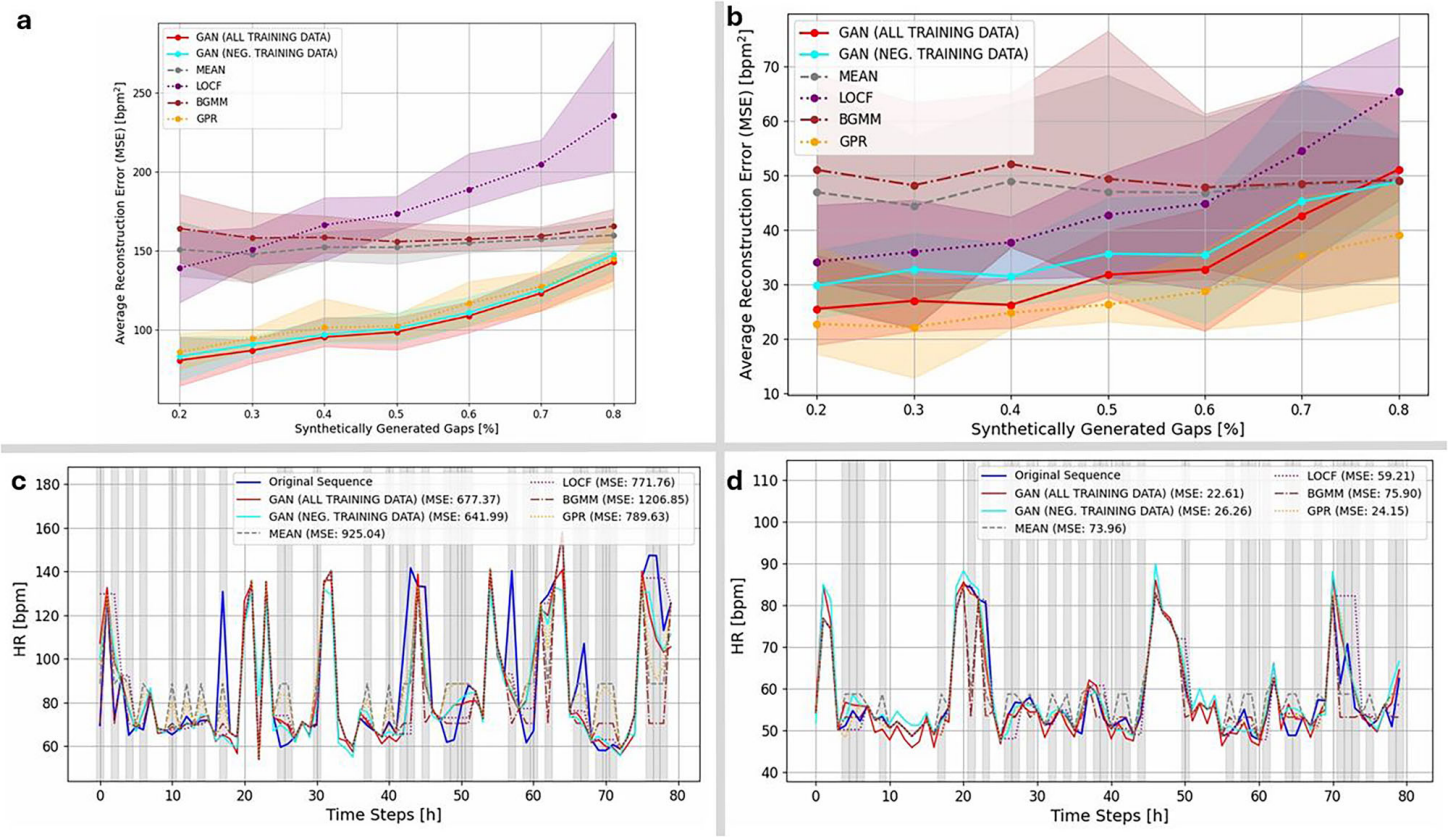
GAN-based imputation outperforms conventional methods in reconstructing wearable heart rate data. **a** COVID-19 cohort; **b** malaria cohort. Mean squared error (MSE) as a function of synthetically generated gap ratio for six imputation methods across 100 uniformly masked sequences per group. GAN models consistently achieve the lowest reconstruction error, particularly at higher missingness levels; in the malaria cohort, Gaussian process regression (GPR) attains a slightly lower mean MSE, but remains within the GAN error margin. Shaded regions indicate the minimum–maximum error range. **c** COVID-19; **d** malaria. Representative heart rate time series comparing reconstructions from each method against the original sequence. GAN-based outputs preserve circadian structure and physiological variability more faithfully than Gaussian process regression (GPR), Bayesian Gaussian mixture models (BGMM), or simpler approaches such as mean imputation and last observation carried forward (LOCF). These results demonstrate the GAN's capacity to restore missing segments while maintaining biologically coherent dynamics critical for downstream anomaly detection.

The advantage of the GAN-based approach was most pronounced in the COVID-19 dataset (Fig. 3a), although Gaussian process regression (GPR) achieved comparable performance within error margins in the malaria cohort (Fig. 3b). In representative examples, the GAN reduced mean squared error (MSE) by 29% in the COVID-19 data (Fig. 3c) and by 58% in the malaria data (Fig. 3d), relative to the average performance of the baseline methods. Models trained on all available data—including infected individuals—outperformed those trained exclusively on non-infected cases, suggesting that broader exposure to physiological variability enhances generalizability. Beyond quantitative improvements, visual inspection of the imputed traces (Fig. 3c, d) confirmed that the GAN more reliably preserved heart rate dynamics and circadian structure—features that are essential for identifying early autonomic disruptions linked to infection onset.

**Fig. 4:**
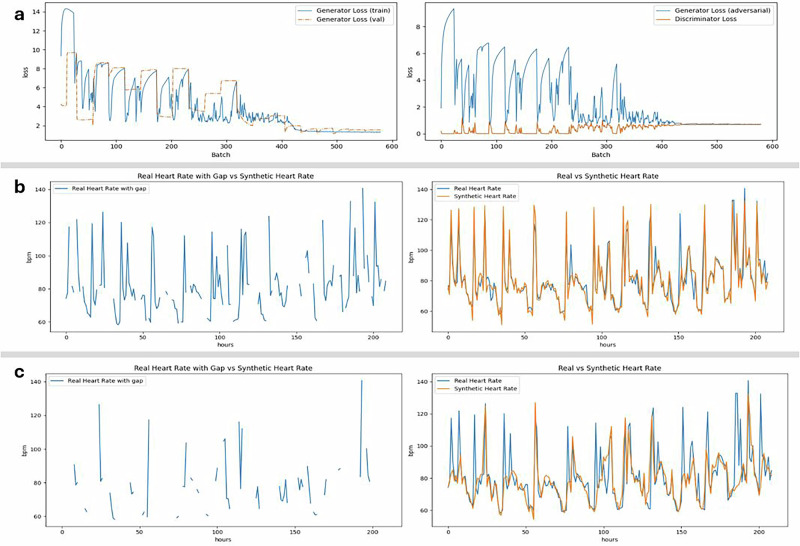
GAN training yields stable reconstructions even under extreme data sparsity. **a** Generator and discriminator losses during training: convergence of both loss curves and smooth adversarial loss progression indicate consistent learning and resistance to overfitting. **b**, **c** Representative heart rate (HR) sequences with 40% (**b**) and 70% (**c**) missingness. Left panels show the original sequences with gaps; right panels compare the real and GAN-reconstructed signals. In both cases, reconstructions preserve circadian structure and physiological variability, with high fidelity even at severe data loss. These results demonstrate the model’s ability to recover biologically coherent dynamics from sparse, noisy biosensor data, supporting its use in downstream health monitoring pipelines.

We further assessed the robustness of the GAN under conditions of extreme data sparsity. As shown in Fig. 4a, the generator maintained high-fidelity reconstructions even when over 70% of the input data was missing. Training dynamics remained stable, with consistent convergence of generator and discriminator losses and a steady decline in adversarial loss, suggesting that the model learned meaningful temporal structure despite limited observable input. Although the GAN was trained exclusively on COVID-19 data, it effectively reconstructed HR trajectories and restored circadian patterns in high-missingness malaria sequences (Fig. 4b, c), indicating its capacity to capture fundamental physiological dynamics rather than overfitting to specific diseases or populations.

**Fig. 5:**
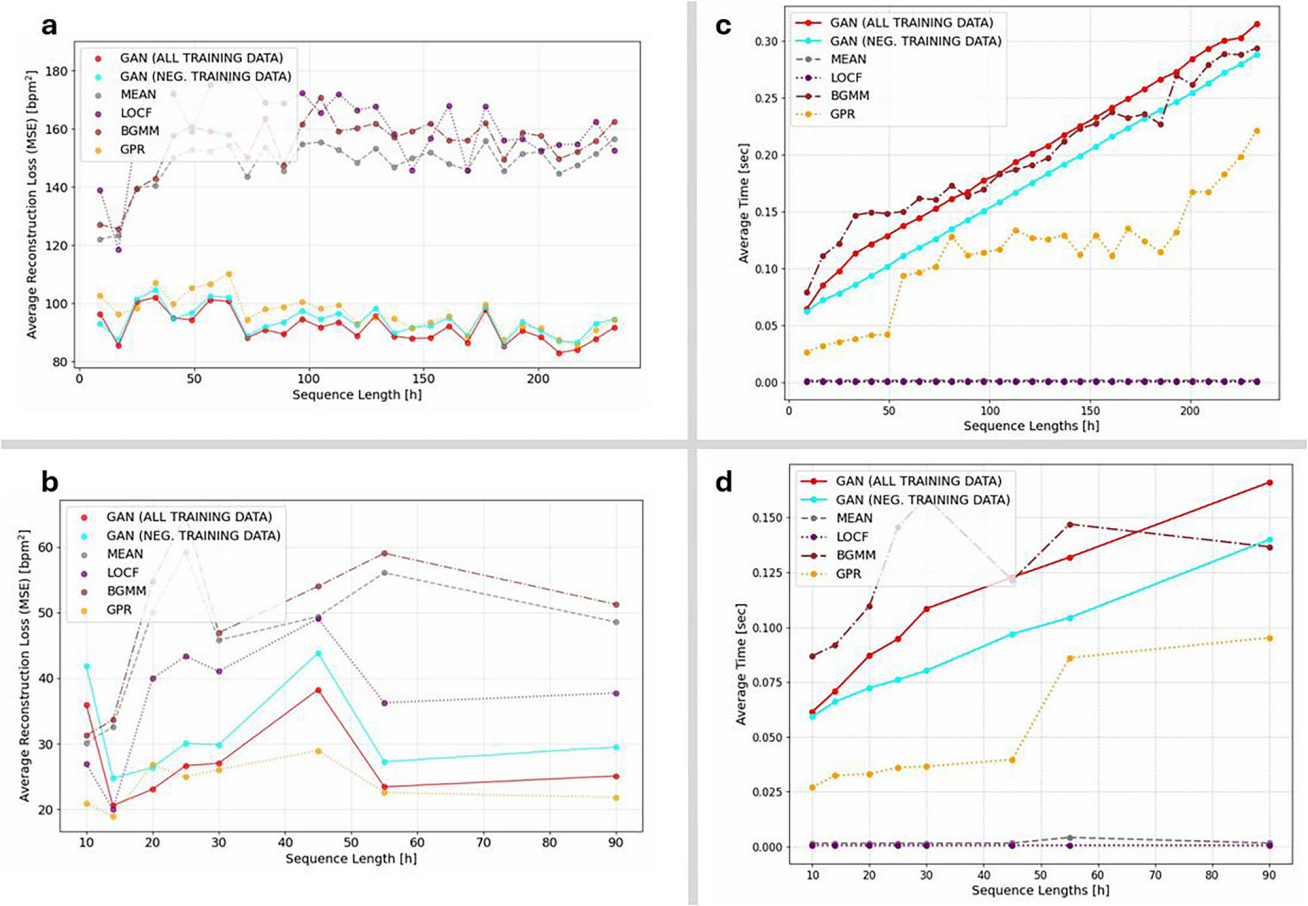
GAN-based imputation delivers high accuracy and efficiency across varying sequence lengths. **a,b** COVID-19 (**a**) and malaria (**b**) cohorts at 40% missingness. Across a wide range of sequence lengths, GAN achieves consistently low reconstruction error (MSE), matching or outperforming all baseline methods; in the malaria cohort, Gaussian process regression (GPR) attains competitive performance at certain sequence lengths but lacks the structural fidelity of GAN reconstructions. **c,d** Average imputation runtime for the same cohorts. GAN scales linearly to longer sequences, with computation times remaining practical for real-time or near-real-time applications. These results demonstrate that the model combines high reconstruction accuracy with computational efficiency, supporting its feasibility for large-scale health monitoring deployments.

Operational feasibility is critical for real-world deployment, particularly in resource-limited settings. Although more recent architectures such as Transformers and diffusion models have demonstrated strong capabilities in time-series applications, they require substantially greater resources during training and inference^23,24^. In contrast, our lightweight GAN-based framework maintained consistently lower mean squared error (MSE) than baseline imputation methods of similar complexity across varying sequence lengths in both cohorts, while preserving biologically plausible temporal patterns (Fig. 5a, b). These improvements were achieved without sacrificing computational efficiency. As shown in Fig. 5c, d, imputation time scaled linearly with sequence length and remained below 0.3 s even for extended sequences—supporting its suitability for edge-computing platforms and real-time use in low-connectivity environments.

### Impact on downstream anomaly detection

To assess whether improved imputation fidelity translated into better infection detection, we evaluated downstream anomaly detection performance using a long short-term memory autoencoder (LSTM-AE). The model was trained to reconstruct overnight HR patterns during presumed healthy periods and to flag anomalies based on elevated reconstruction error. We compared performance across two input types—raw (non-imputed) data and GAN-imputed sequences—using both the malaria and COVID-19 cohorts.

When applied to the raw data, the LSTM-AE achieved a recall of 86% and an optimized F1-score of 64%. With GAN-imputed data, recall increased to 89.45% and precision rose to 69.75%, yielding an F1-score of 78.38%. This improvement likely occurred because the GAN filled in missing data with patterns that were less similar to healthy ones, making signs of infection stand out more clearly. As a result, the number of infected sequences increased, shifting the balance between healthy and infected data from 0.92:1 to 0.46:1. Full evaluation metrics are presented in Section “Evaluation metrics”.

While GAN-imputed data improved LSTM-AE performance, the model’s interpretability remained limited and its outputs were still sensitive to potential biases from imputation. To address these challenges, we adopted a finite state machine (FSM) anomaly detection algorithm originally published by Alavi et al.^2^. The FSM relies on rule-based transitions between discrete physiological states and does not require training on imputed sequences, reducing the risk of data leakage. Its rule-based structure provides greater interpretability and clinical caution.

**Fig. 6:**
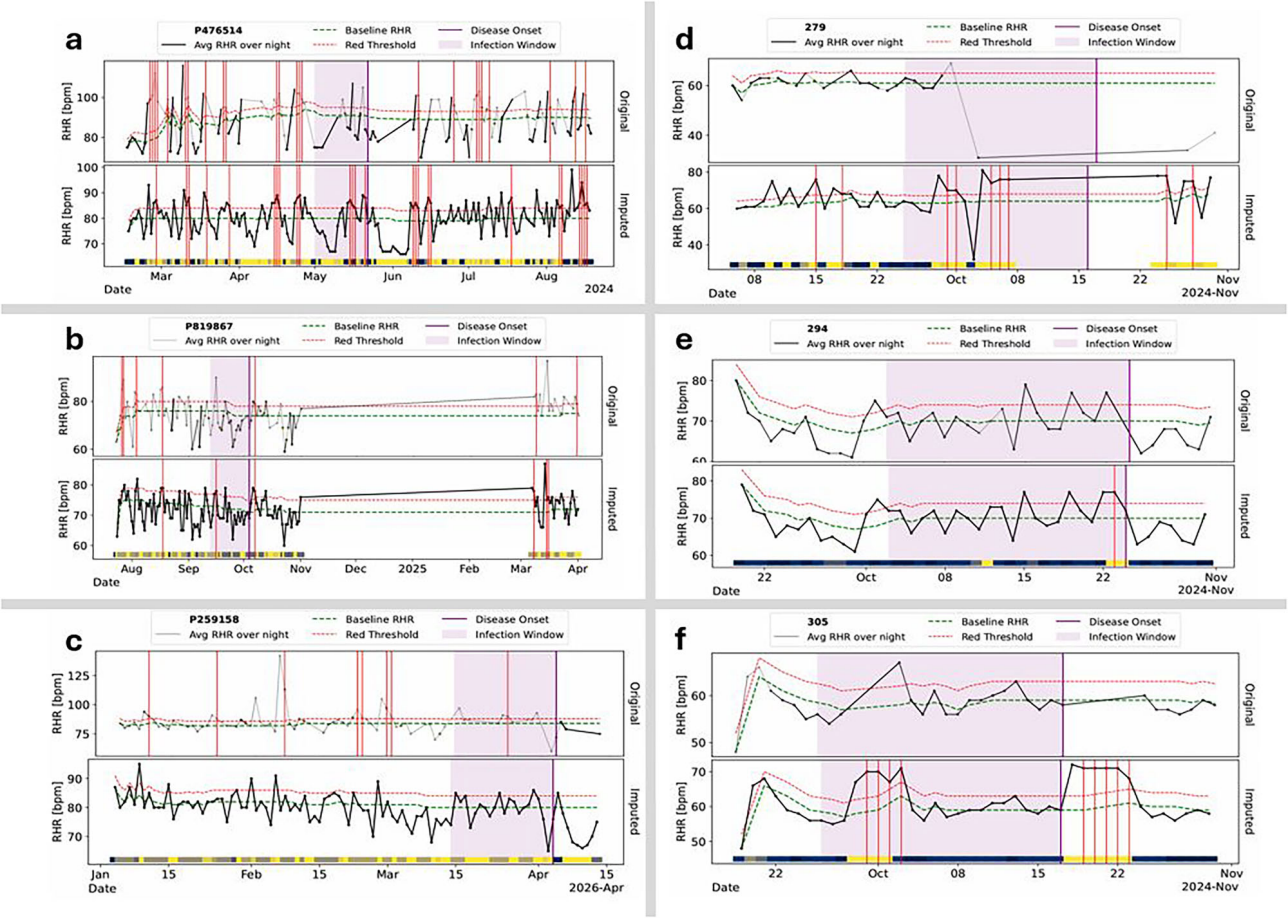
GAN-imputed signals enable reliable anomaly detection of infection onset. **a**, **b** COVID-19 cohort examples where the finite state machine (FSM) fails to detect infection from raw RHR data (purple window), but GAN-based imputation triggers timely alerts (red) that align with PCR-confirmed diagnoses while removing prior false positives. Color intensity in the imputed traces reflects the proportion of GAN-generated data per night, adding interpretability. **c** A rare missed COVID-19 case under extreme sparsity: threshold crossing in raw data occurs by chance, while the GAN reconstruction smooths the subtle deviation, attenuating the anomaly. **d**–**f** Malaria cohort examples show accurate infection detection without false alerts, even in severely gapped sequences. Across both cohorts, robust imputation restores biologically meaningful dynamics, improving the fidelity and specificity of wearable-based anomaly detection.

This decision was supported by case-level qualitative assessments. In one example (Fig. 6a, b), the FSM failed to detect an infection using raw data but successfully triggered an alert when GAN-imputed signals were used—closely matching the timing of a PCR-confirmed diagnosis. Conversely, in a rare counterexample (subject P259158; Fig. 6c), the anomaly was detected using raw data but was missed after GAN-based imputation. In this case, nightly HR averages were smoothed below the FSM’s alert threshold, effectively masking the infection signal despite confirmed disease.

This observation highlights a key trade-off in imputation-based approaches: while improved fidelity can reduce false positives, over-smoothing may suppress critical variance that signals disease onset. Across the cohort, GAN-based imputation reduced the false positive rate by 2%, lowering it to 7.7%. This modest improvement may enhance user trust, reduce alarm fatigue, and increase adherence to long-term monitoring. Nevertheless, the occasional missed detection—especially in clinically vulnerable or under-monitored populations—emphasizes the importance of retaining local diagnostic variance as the proportion of imputed values increases. A practical safeguard is to accompany each reconstructed segment with an imputation-density indicator, highlighting intervals where reconstruction confidence is lower. In such cases, inspection of the corresponding raw signal may be warranted to ensure that clinically relevant deviations—such as the counterexample observed in this cohort—are not overlooked.

### Generalization to low-resource settings

The ability to generalize across contexts is particularly important for low- and middle-income countries (LMICs), where limited local datasets, infrastructure constraints, and the need for rapid deployment often preclude the development of setting-specific models. Local data are frequently constrained in size, duration, or diversity, limiting the feasibility of training disease-specific algorithms on in-country populations. Despite these challenges, the GAN model—originally trained on COVID-19 data—maintained stable reconstruction fidelity when applied to the malaria sequences without retraining (see Section “Fidelity of GAN-based imputation”). This finding supports the feasibility of using pretrained generative models as foundational digital infrastructure for infection monitoring in data-scarce environments.

Importantly, malaria produces characteristic temporal HR dynamics—such as fever-induced tachycardia correlated with parasitemia cycles—that are distinct from the inflammatory and injury-mediated tachycardia seen in COVID-19^25,26^. Our analyses suggest that these differences underline the ability of the GAN to adapt to divergent temporal patterns across diseases.

The GAN’s ability to accommodate these physiological differences suggests that it captures higher-order temporal features—such as recovery slopes and nocturnal troughs—that are conserved across infectious disease contexts. In the malaria cohort (Fig. 6d–f), GAN-based imputation enabled the detection of anomalies that were previously obscured by missing data. This underscores the signal-enhancing capacity of imputation: rather than merely filling gaps, the GAN restored latent physiological deviations associated with infection. Across the cohort, 100 malaria episodes were detected, 42 of which were identified exclusively due to GAN-enabled imputation (see Supplementary Data 4).

Beyond binary detection accuracy, the temporal persistence of anomaly alerts is important for evaluating alignment with diagnostic protocols. In malaria-endemic settings, current U.S. Centers for Disease Control and Prevention (CDC) guidelines recommend repeated blood smear microscopy every 12-24 hours over three time points before excluding a diagnosis^27^, due to intermittent parasitemia and initially low parasite loads. In our cohort, the average alert duration during early infection extended to 3.5 consecutive days with GAN-based imputation, compared to 2.8 days without it (see Supplementary Data 4). This extended alert span provides objective evidence that GAN-imputed signals can enhance the temporal continuity of physiological deviations relevant for diagnosis.

Two illustrative examples (Participants 279 and 305; see Section GAN-Only Early Alerts in Malaria Cases) showed sustained early alerts that temporally overlapped with the diagnostic cadence used in microscopy protocols. These cases provide further evidence that GAN-based imputation preserves infection-related temporal structure that would otherwise be obscured by data loss.

### GAN-only early alerts in malaria cases

Early recognition of malaria is essential in endemic regions, where timely treatment can reduce morbidity and transmission. To assess whether GAN-based imputation improvements translated into earlier, clinically actionable alerts, we focused on 42 participants from the Kenyan malaria cohort. These individuals had confirmed symptomatic malaria infections, complete symptom diaries, and received anomaly alerts only after GAN-based imputation. In 36 of these 42 cases (86%), the system generated presymptomatic alerts—defined as anomaly flags occurring at least 72 h before symptom onset (see Supplementary Data 4)—a time window considered actionable in malaria surveillance programs^28^.

A representative case is Participant 279, who received a malaria diagnosis on October 16, 2024 (Fig. 6d). Only seven HR datapoints were recorded in the three weeks prior to symptom onset—insufficient for detection using raw data. After GAN-based reconstruction, the time series revealed a progressive elevation in nightly heart rate and loss of circadian amplitude beginning 16 days before symptom onset. The first anomaly alert coincided with the participant ceasing device use—behaviorally linked to symptom onset in digital phenotyping^29^. Despite this adherence gap, the GAN-reconstructed sequences enabled four additional alerts over the next 5 days, all preceding diagnosis and high-fever onset.

Structured symptom tracking began on September 12, 2024 (see Supplementary Data 3). Initial reports were likely inflated due to first-entry effects^30^, but stabilized by week two. Mild symptoms, such as fatigue and headache, were recorded on days flagged as presymptomatic by the anomaly detector. Diagnostic confirmation occurred in week six, followed by escalating symptom burden, consistent with malaria’s paroxysmal progression^25^. During temporary symptom relief in weeks five and seven, no GAN alerts occurred, suggesting specificity to physiologically coherent deviations. Under a hypothetical early-intervention scenario, GAN alerts in weeks three or four could have enabled timely diagnosis and treatment—potentially reducing 86% of this participant’s total symptom burden (49 of 57 symptoms).

A second example, Participant 305 (Fig. 6f), illustrates similar presymptomatic alerting. The first alert occurred on September 30, 2024, 17 days before a confirmed malaria diagnosis on October 17. Raw data were sparse, but GAN-imputed sequences revealed sustained nocturnal HR elevation and blunted circadian rhythm—yielding four alerts prior to diagnosis. Symptom tracking began after diagnosis and revealed a febrile trajectory with persistent systemic symptoms. A second cluster of five GAN alerts aligned with this post-diagnosis phase, suggesting the system’s potential to track ongoing deterioration.

In contrast, Participant 294 (Fig. 6e) received a late-stage alert less than 24 hours before confirmed diagnosis. While this validated model responsiveness, the short lead time limited clinical utility.

These case-level observations are supported by cohort-wide patterns. Among the 36 participants with presymptomatic alerts, an estimated 65% of symptoms in 27 individuals could have been prevented or substantially mitigated under ideal early-intervention scenarios (see Supplementary Data 3). Because all early alerts were dependent on GAN-based imputation, these findings reframe missing data not as a diagnostic barrier but as a recoverable signal. By restoring physiologically meaningful structure, this approach offers a scalable strategy for advancing malaria detection from reactive to preemptive—particularly in high-burden, low-resource settings where timing is critical.

In the 42 malaria cases where alerts were enabled exclusively by GAN-imputed data, the system provided a median lead time of 11.9 days before symptom onset (see Supplementary Data 4). Notably, this duration aligns closely with the 11.7-day interval reported in controlled human malaria infection (CHMI) studies as the average time from inoculation to detectable blood-stage parasitemia—the diagnostic gold standard for malaria^31^. This concordance, achieved without prior exposure to malaria-specific data, suggests that the GAN-based model did more than reconstruct missing values; it learned to preserve physiologically meaningful features indicative of early infection, generalizing across distinct disease contexts.

## Discussion

Our findings demonstrate that generative imputation using GANs can enhance the continuity, interpretability, and diagnostic value of physiological data from wearable devices, particularly in LMICs where high data loss is common. By reconstructing missing HR sequences in two distinct disease cohorts, the model enabled early detection of infection-related anomalies that would have otherwise remained undetected. In the malaria cohort, alerts from GAN-imputed data achieved median lead times aligned with gold-standard parasitemia windows, despite the model being trained exclusively on COVID-19 data. This cross-disease generalization highlights the potential of pretrained generative models to support scalable surveillance across distinct febrile illnesses in data-scarce environments.

Early detection of infectious diseases through wearable-derived anomaly alerts offers significant clinical advantages, with malaria being a prime example, where presymptomatic intervention can substantially reduce morbidity and transmission^32–34^. Studies of community-based malaria programs have shown that timely treatment within 48-72 hours of fever onset markedly reduces parasite burden and secondary cases^35,36^. In our study, GAN-based imputation enabled alerts up to two weeks before symptom onset—closely matching the parasitemia detection cadence in CDC microscopy guidelines^27^ and paralleling timelines reported in CHMI trials^31^. Such early-warning capacity is especially critical in regions where health system delays or care-seeking barriers impede timely diagnosis. Integrating algorithmic alerts into community health worker (CHW) workflows could enhance triage, reduce symptom burden, and improve resource allocation^37^. They could also inform planning for outreach teams and diagnostic deployment by non-governmental organizations (NGOs)^38^.

As digital health infrastructure expands across Africa, wearable-based detection systems may help shift the paradigm from reactive care to anticipatory surveillance by enabling real-time, individualized risk monitoring at scale. In practice, CHWs equipped with mobile-linked wearables could receive automated alerts from continuously tracked physiological signals—prompting timely home visits or diagnostics before symptoms escalate. By delivering feedback, wearables can empower individuals to recognize early physiological changes and adjust behaviors—transforming them from passive data subjects into active participants in their own health management. At the community and population level, aggregated data could then feed into regional dashboards to inform outbreak response while guiding personalized, daily decisions. This multilevel integration bridges the gap between population-level screening and individualized clinical action—offering a scalable foundation for more equitable and proactive care in African health systems.

Beyond individual detection, our approach contributes to a broader vision of precision public health in Africa—leveraging real-time, individualized physiological data to guide public health action in resource-limited settings. The demonstrated cross-context generalization of GANs between distinct infectious diseases without retraining highlights their potential as scalable infrastructure, particularly where labeled data are scarce. In these contexts, pretrained models can serve as foundational tools for adaptive triage and early outbreak detection. Realizing this potential requires robust implementation strategies, including support for local capacity building, interoperable systems, and ethical frameworks that promote transparency, trust, and sustainability.

Consistent with this vision, strong evidence of cross-disease generalizability emerged in the malaria cohort. Despite being trained exclusively on COVID-19 data, the system generated anomaly alerts in 42 malaria cases with a median lead time of 11.9 days—closely matching the 11.7-day parasitemia window reported in controlled human infection studies. This temporal concordance suggests the model internalized general physiological signatures of infection progression rather than pathogen-specific features. As a result, generative imputation is repositioned from a preprocessing step to a biologically informative inference tool—capable of recovering latent disease trajectories even with extensive data gaps. This signal-enhancing capability elevates GANs from data-repair utilities to generators of latent clinical signals, representing a meaningful advance in the extraction and deployment of digital biomarkers from real-world wearable sensor data.

Building on this ability, this work introduces a technically novel framework that integrates generative imputation with interpretable, rule-based anomaly detection—offering a robust solution for infection monitoring from sparse wearable data. Unlike traditional imputation methods such as mean substitution, last observation carried forward, or even probabilistic models like Gaussian processes^39^, which often oversmooth clinically relevant variability or assume stationary signal properties, our GAN-based approach more faithfully captures dynamic, biologically relevant patterns—such as diurnal rhythms, recovery slopes, and heart rate volatility—critical for detecting early autonomic deviations associated with infection^40–42^. By reconstructing not just plausible but pathophysiologically relevant sequences, the model enhances downstream detection and enables timely alerting.

However, several limitations of this study should be acknowledged. First, while GAN-based imputation enhanced signal continuity and early detection, it may introduce bias through over-smoothing, potentially suppressing subtle yet clinically important anomalies—particularly in low-prevalence or atypical cases. Second, the analysis relied on univariate heart rate data, limiting the ability to capture broader physiologic responses; integrating biosignals such as skin temperature, respiratory rate, or activity could improve detection fidelity and clinical interpretability. Third, the study was retrospective, and while it leveraged real-world wearable data, prospective validation in field settings is needed to evaluate usability, trust, and effectiveness.

Resource constraints led to lower adherence and data availability in the malaria cohort compared to the COVID-19 cohort, which had daily wear-time and synchronized uploads. As a result, this dual-cohort design allowed evaluation of model generalizability and real-world performance under contrasting conditions of data richness, testing frequency, and infrastructure limitations. Nonetheless, these cohorts represent only the specific geographic regions studied, and broader validation across diverse epidemiological, demographic, and health system contexts is required to confirm equity and performance. Furthermore, because malaria transmission is vector-borne and influenced by environmental factors such as temperature, humidity, and rainfall, future implementations could integrate geospatial and climatic data to improve sensitivity to outbreak precursors.

Looking ahead, prospective deployment of this system in real-world settings will be critical to validate its clinical and operational utility. Integrating real-time alerting into community health workflows—particularly in low-resource contexts—could enable dynamic, decentralized responses to early signs of infection. Wearable-based monitoring is especially relevant in LMICs, where health systems often face limitations in both the quantity and quality of care delivery. By enabling continuous, non-invasive physiological tracking outside clinical facilities, wearable systems can serve as frontline tools for early triage and targeted intervention.

Future iterations should incorporate multimodal biosignals—such as skin temperature, respiratory rate, heart rate variability, and oxygen saturation—many accessible through commercial wearables. Emerging technologies, such as wearable sweat sensors, offer promise for continuous, non-invasive monitoring of biochemical markers like glucose, cortisol, or electrolytes, which could inform hydration status, metabolic shifts, and inflammatory responses. For vector-borne diseases like malaria, integrating environmental data alongside physiological signals may improve prediction accuracy, given the dependence of mosquito vector activity on climatic conditions. This dual-layered approach—capturing both host and environmental dynamics—could support more granular, context-sensitive surveillance frameworks in endemic regions.

While malaria serves as a proof-of-concept, the approach is extensible to other infectious and non-communicable diseases (NCDs) such as cardiovascular or metabolic disorders. The ability to detect physiological deviations early positions wearables as key tools in the evolution toward precision public health and telemedicine. By enabling personalized, remote monitoring, these systems could strengthen chronic disease management, reduce care delays, and improve access to preventative care.

In conclusion, this work reframes missing data from wearable sensors not as a barrier, but as a solvable challenge in the advancement of digital health. By applying generative adversarial networks for physiological imputation, we demonstrate that fragmented time series can yield actionable clinical insights—enabling earlier detection of infection and improving alignment with diagnostic windows. This approach positions GAN-based imputation as a foundational component of scalable, equitable digital diagnostics, especially in settings where diagnostic delays disproportionately impact outcomes.

By restoring latent physiological structure and enabling personalized, real-time monitoring, our system supports a shift from reactive care to preemptive action. Wearables can thus serve as instruments for early triage, decentralized health delivery, and proactive surveillance. As digital health infrastructure expands globally, such approaches will be essential to achieving more inclusive, adaptive, and precision-driven healthcare.

## Methods

### Study design and participants

This study evaluated the capacity of generative adversarial networks (GANs) to impute missing heart rate (HR) data collected from wearable devices and assessed how such imputations affect downstream anomaly detection in infectious disease monitoring. Two distinct cohorts were used to assess performance across varied environmental, clinical, and technological contexts.

The COVID-19 cohort included 3318 participants, and a subset of 2124 participants was made available from a previously published surveillance study, where participants wore commercially available devices to continuously record biometric data including heart rate and step count. Further details on data collection procedures and ethical approval can be found in the original publication^2^.

The malaria cohort consisted of 300 adults aged 19-82 residing in the Siaya Health and Demographic Surveillance System (HDSS), a population-based research platform in rural Western Kenya that continuously collects demographic and health data^43^; of these, only 290 participants had usable data. Participants were recruited through the Akala healthcare facility, a high-volume clinic with qualified personnel, and monitored using Garmin Vivosmart 5 wearable devices. Malaria infection status was assessed every three weeks using WHO-compliant rapid diagnostic tests (RDTs) and participant symptom diaries, which were blindly collected during regular field worker home visits. Physiological data (heart rate, steps, sleep) were retrieved every 5–7 days by study staff, who also managed device synchronization and charging.

Our mixed-methods observational study received ethics approval from the Research Ethics Committees at the Kenya Medical Research Institute, Nairobi (approved on 23 October 2023; SERU 4826), and Heidelberg University Hospital, Germany (approved on 14 February 2023; S-824/2022). Written informed consent was obtained from all participants prior to enrollment, with data handled in accordance with Kenyan and German data protection regulations.

### Data preprocessing

All wearable data were time-stamped and collected in a continuous streaming format. As illustrated in Supplementary Fig. 1, preprocessing began with integrity checks to exclude corrupted segments, full-day dropouts, and physiologically implausible heart rate (HR) values (e.g., < 30 bpm). HR signals were resampled to a one-minute resolution and aligned with step count data to form a multivariate time series for imputation and anomaly detection.

Nightly resting heart rate (RHR) was calculated as the median HR between midnight and 07:00 hours, minimizing behavioral confounders. Days with fewer than four valid hours in this window were excluded from anomaly detection and imputed via the GAN. Longitudinal baselines were computed per participant using the mean nightly RHR over the first 28 valid days and updated on a rolling basis. Where available, step-based inactivity markers were included as auxiliary inputs.

To evaluate sensitivity to motion filtering, we tested varying inactivity thresholds. A 12-min inactivity window reduced false positives but suppressed early anomaly detection. To preserve sensitivity to early signals, we adopted a 0-min threshold across cohorts, accepting moderate noise to maximize detection fidelity. Additional evaluation details are provided in Section “Evaluation metrics”.

### Data splitting and sequence generation

To train the autoencoder (AE) anomaly detection model, we applied subject-level data splitting to prevent inter-subject leakage. Twenty percent of participants were held out as an independent test set. The remaining subjects were randomly split into training (90%) and validation (10%) sets.

For the AE, continuous HR recordings were segmented into 49-h sliding windows spanning seven consecutive nights (7 h/night), designed to capture stable individual baselines^44^. The stride was set to 24 h, creating overlapping sequences that shifted by 1 day. This configuration enabled the model to learn weekly temporal dynamics, including circadian rhythms and potential recovery trajectories.

For the GAN-based imputation model, we employed a flexible sampling strategy. Variable-length sequences of 8*n* + 1 h (*n* ∈ {1, …, 29}) were extracted from continuous, gap-free intervals. Start times were spaced 241 h apart to minimize overlap and enhance circadian diversity. Each sequence was corrupted with a binary mask *m* ∈ {0, 1}^*T*^, where *m*_*t*_ = 0 denotes a missing value at time *t*, introducing 40–70% artificial dropout. This encouraged the model to learn both short- and long-range temporal dependencies. GAN training was performed on hourly HR averages using an 80%–10%–10% COVID-19 cohort subject-level split.

These sequences served as input for the AE and GAN models (see Supplementary Fig. 2), providing a modular, biologically structured framework adaptable to alternative input resolutions, target conditions, or model architectures.

### GAN architecture and training

The GAN architecture used for imputing missing HR values is shown in Supplementary Fig. 3. Each input consisted of a partially observed HR sequence and a corresponding binary mask vector (see Section Data Splitting and Sequence Genera tion)) identifying the missing values.

The generator *G* followed a U-Net-style design comprising three 1D convolutional layers for local feature extraction, followed by three bidirectional LSTM layers to model long-range temporal dependencies. A final time-distributed dense layer produced a fully reconstructed sequence. The discriminator *D* was implemented as a convolutional network and used adversarial loss to evaluate the realism of temporal patterns within the imputed output.

To ensure that generated outputs were both realistic and accurate, the model was trained using a composite objective. The generator loss *L*_*G*_ combined an adversarial component *L*_adv_, which encouraged naturalistic reconstructions, with a root mean squared error (RMSE) term *L*_RMSE_ that penalized deviations from ground truth at masked time points:

1 $${L}_{{\rm{adv}}}=-{{\mathbb{E}}}_{x \sim {p}_{{\rm{data}}}(x)}\left[\log D(G(x,m))\right],$$

2 $${L}_{{\rm{RMSE}}}=\sqrt{\frac{1}{| M| }\mathop{\sum }\limits_{t\in M}{({x}_{t}-{\widehat{x}}_{t})}^{2}},$$

3 $${L}_{G}={L}_{\mathrm{adv}}+{\rm{\lambda }}\cdot {L}_{\mathrm{RMSE}},$$

where *x* denotes the original sequence, *G*(*x*, *m*) is the generator output, *M* is the set of masked time steps, ${\widehat{x}}_{t}$ is the reconstructed value, and *λ* balances realism and reconstruction accuracy.

The discriminator was trained using binary cross-entropy loss:

4 $${L}_{D}=-{{\mathbb{E}}}_{x \sim {p}_{{\rm{data}}}(x)}\left[\log D(x)\right]-{{\mathbb{E}}}_{x \sim {p}_{{\rm{data}}}(x)}\left[\log \left(1-D(G(x,m))\right)\right].$$

The entire training process can be viewed as a minimax game between generator and discriminator^17^:

5 $${L}_{{\rm{GAN}}}=\mathop{\min }\limits_{G}\mathop{\max }\limits_{D}\,{{\mathbb{E}}}_{x \sim {p}_{{\rm{data}}}(x)}\left[\log D(x)\right]+{{\mathbb{E}}}_{x \sim {p}_{{\rm{data}}}(x)}\left[\log \left(1-D(G(x,m))\right)\right].$$

Unlike conventional GANs that aim to generate synthetic data from random noise, our model focused on reconstructing partially observed physiological sequences. The RMSE loss was essential in this setting, directly anchoring the generator to available ground truth and stabilizing training^45^. Without it, adversarial training alone risked unrealistic outputs or mode collapse.

To improve robustness under increasing levels of missingness, we employed curriculum learning^46^: training began with masking rates of 40% and gradually increased to 70% over successive epochs, mimicking progressively more difficult imputation scenarios. Optimization was performed using the Adam optimizer (learning rate = 0.001), and early stopping was applied to avoid overfitting.

Although the model was trained solely on data from the COVID-19 cohort, it demonstrated strong generalization to the Kenyan malaria cohort. This cross-cohort performance suggests the model can reliably reconstruct incomplete wearable sensor data in diverse real-world settings—supporting its potential utility in longitudinal health monitoring, early disease detection, and digital surveillance in low-resource environments.

### Autoencoder-based anomaly detection

To benchmark GAN-based imputation against standard machine learning approaches^47,48^, we implemented a deep bidirectional LSTM autoencoder (AE) for unsupervised anomaly detection, shown in Supplementary Fig. 4. The model was trained exclusively on HR sequences from participants without confirmed infections, allowing it to learn individual baselines and identify deviations as potential anomalies.

The AE architecture consisted of three 1D convolutional layers for feature extraction, followed by multiple bidirectional LSTM layers for temporal modeling. A bottleneck layer enforced compression, and the decoder reconstructed the sequence via a repeat vector, additional LSTM layers, and a time-distributed dense output layer. Input sequences are described in Section “Data splitting and sequence generation”.

The training objective was to minimize a composite reconstruction loss combining mean squared error (MSE), negative Pearson correlation, and Huber loss:

6 $$L=\,\mathrm{MSE}+{{\rm{\lambda }}}_{1}\cdot {L}_{\mathrm{Pearson}}+{{\rm{\lambda }}}_{2}\cdot {L}_{\mathrm{Huber}},$$

where the Pearson term preserved temporal alignment and the Huber loss added robustness to outliers. Model hyperparameters—including loss weights, bottleneck size, and learning rate—were optimized via grid search. A full list of model configurations, loss functions, and training parameters is provided in Supplementary Tab. 1.

Anomaly thresholds were determined using a range of strategies^49^, including z-score cutoffs, receiver operating characteristic (ROC) curve analysis, Youden’s *J* statistic^50^, and one-class support vector machines (SVMs). These methods enabled fine-tuning of sensitivity and specificity, particularly in the presence of GAN-imputed sequences (Supplementary Fig. 5), with resulting performance summarized in Table 1.

**Table 1:** Comparative performance evaluation for the LSTM-AE pipeline on the malaria cohort

LSTM-AE performance evaluation (malaria cohort)						
Input	Sensitivity	Specificity	Precision	Accuracy	F1-score	ROC AUC
Raw	86.00%	12.00%	51.00%	50.00%	64.00%	53.00%
GAN-imputed	**89.45%**	**16.35%**	**69.75%**	**66.29%**	**78.38%**	**57.67%**
Difference	+3.45%	+4.35%	+18.75%	+16.29%	+14.38%	+4.67%

Best-performing values for each metric are shown in bold.

### FSM-based anomaly detection

In contrast to machine learning-based anomaly detection, we implemented a rule-based finite state machine (FSM) to detect significant nightly RHR deviations. The NightSignal algorithm^2^, originally developed for wearable-based illness detection, issued alerts when nightly RHR exceeded a threshold of 4 beats per minute for two consecutive nights, relative to a 28-day moving baseline.

This approach relies on fixed rule transitions and is purposefully simpler than LSTM-based models, offering a transparent and interpretable benchmark across both cohorts. We used it to compare the performance of GAN-based imputation under clinically motivated anomaly detection criteria. Further methodological details are available in the original publication^2^. Performance results are presented in Table 2.

**Table 2:** Comparative performance evaluation for the NightSignal pipeline on the malaria cohort

NightSignal performance evaluation (malaria cohort)						
Input	Sensitivity	Specificity	Precision	Accuracy	F1-score	Balanced accuracy
Raw	35.53%	**96.43**%	**60.38**%	**88.35**%	44.73%	65.98%
GAN-imputed	**50.88**%	92.52%	51.03%	87.00%	**50.95**%	**71.70**%
Difference	+15.35%	−3.91%	−9.35%	−1.35%	+6.22%	+5.72%

Best-performing values for each metric are shown in bold.

### Evaluation metrics

For anomaly detection, we computed sensitivity, specificity, precision, accuracy, F1-score, balanced accuracy, and the area under the receiver operating characteristic curve (ROC AUC). These metrics were calculated for both the raw and GAN-imputed sequences, using thresholds determined through global ROC curves (Supplementary Fig. 5) as well as per-subject calibration strategies. Given the well-known trade-off between sensitivity and specificity in classification tasks, we optimized for F1-score as the most balanced evaluation metric.

Table 2 summarizes comparative results for the NightSignal pipeline on the malaria cohort. Performance was evaluated using a day-level definition: a participant was considered correctly identified if at least one anomaly was detected within the 21-day presymptomatic window preceding a RDT-confirmed malaria diagnosis. This formulation accounts for the paroxysmal nature of malaria-related physiological changes. Alerts occurring outside presymptomatic windows were classified as false positives. Because true and false positives are defined on different temporal units, standard confidence interval estimation was not applied; accordingly, results are presented as comparative indicators of relative performance rather than absolute estimates. Additional details regarding metric computation are provided in Supplementary Data 4.

GAN-based imputation substantially increased the coverage of signals, thereby expanding the opportunity space for anomaly detection. As a result, the imputed NightSignal pipeline generated a higher number of alerts overall compared to raw sequences. This shift explains why standard metrics—which favor non-alerting behavior under sparse observation—do not fully capture the clinical value of imputation under denser data availability, as increased diagnostic cadence is not rewarded beyond a single case-level detection.

In addition to day-level performance, we therefore evaluated NightSignal at the relevant case-level, reflecting whether an individual at risk was flagged at least once during the presymptomatic window. Using GAN-imputed input, the F1-score increased from 52.9% to 73.3% (+20.4%), thereby improving the coverage of detected infections.

We conducted an analogous evaluation for the LSTM-AE-based anomaly detection pipeline, observing consistent improvements under GAN-based imputation.

The LSTM-AE exhibits a higher F1-score performance than NightSignal, indicating that GAN-based imputation consistently improves detection across both simple and complex models. LSTM-AE, therefore, provides a useful upper-bound reference for achievable detection performance under imputation.

To assess the impact of signal preprocessing, we also evaluated how different definitions of resting heart rate (RHR) influenced anomaly detection accuracy in wearable data (Supplementary Fig. 6). These results directly informed the choice described in Section “Data preprocessing” to retain a 0-min inactivity threshold, thereby preserving early indicators of physiological change.

### Visualization methods and reproducibility

Most figures were generated using the Matplotlib package in Python. Reproducibility was ensured by fixing random seeds across TensorFlow, NumPy, and Python environments. All GANs and autoencoders were trained on NVIDIA A100 GPUs, which facilitated efficient model training.

## Supplementary information

Supplementary Information

Supplementary Data 1

Supplementary Data 2

Supplementary Data 3

Supplementary Data 4

## Data Availability

De-identified raw heart rate and resting heart rate data supporting the findings of this study have been deposited in a Figshare repository under a CC BY 4.0 license and are available at 10.6084/m9.figshare.31444399. The code supporting the findings of this study is available in a GitHub repository at https://github.com/jwallukhd/gan-imputation-wearable-data.
