# Supplementary material for: Overcoming Data Loss in Wearable Disease Detection with GAN-Based Imputation
Source: NPJ Digit Med. 2026 Mar 27;9:275. doi: 10.1038/s41746-026-02518-4 (PMC13043753; doi:10.1038/s41746-026-02518-4)
Supplement: Supplementary file 1 — Supplementary Information [file 41746_2026_2518_MOESM1_ESM.pdf]

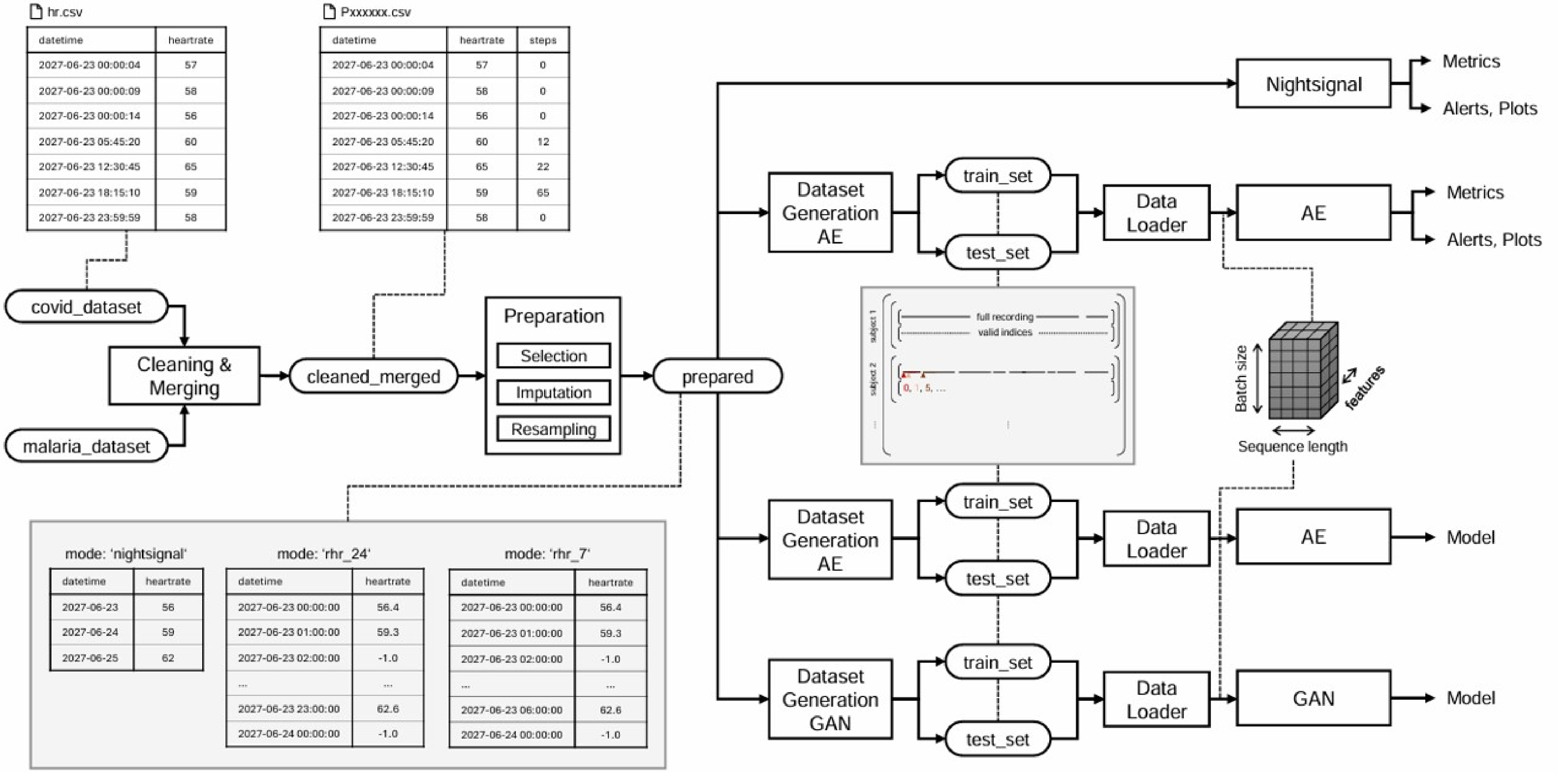

Supplementary Fig. 1: **System architecture for wearable data imputation and infection signal recovery.** A modular pipeline transforms raw wearable records into clinically relevant signals through linked stages of cleaning/merging, preparation (selection, imputation, resampling), dataset generation, and model training. GAN-based imputation operates in one branch, while an autoencoder (AE) anomaly detector processes both raw and imputed sequences for infection onset detection. Outputs feed into Nightsignal and other evaluation modules to assess both anomaly detection performance and signal retention. The architecture supports disease-agnostic inputs, allows flexible preprocessing modes for metrics such as resting heart rate (RHR; see Supplementary Fig. 2), ensures reproducibility via clear abstraction layers, and incorporates explainability—such as proportion-of-imputed-data shading in reconstructed signals (Fig. 6)—to aid interpretation. Designed for adaptability, it applies to both controlled and real-world datasets.

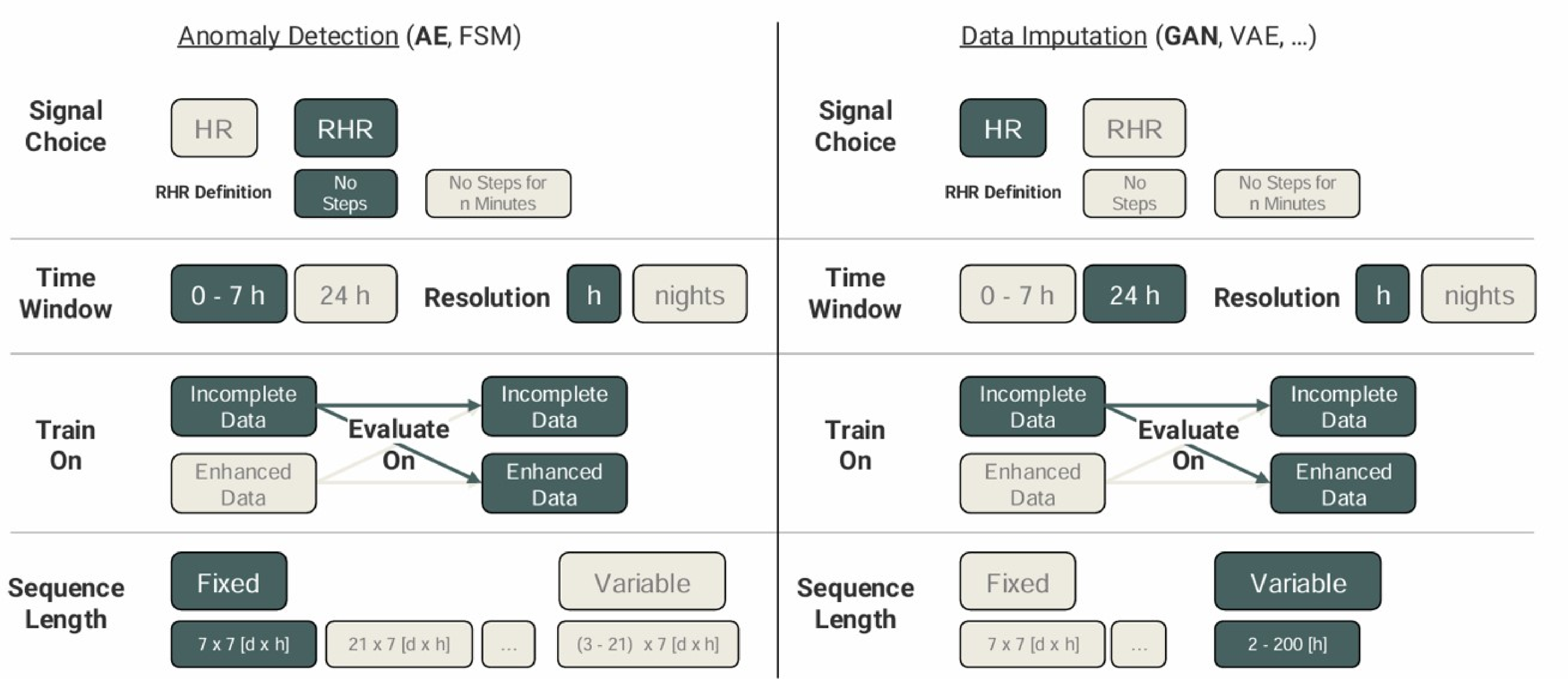

Supplementary Fig. 2: **Modular experimental configurations for anomaly detection and data imputation across physiological data streams.** Configurable pipeline parameters used to set up model training and evaluation, enabling systematic benchmarking of detection and reconstruction tasks. Building on the architecture in Supplementary Fig. 1, this figure specifies adjustable components. **Left (anomaly detection):** The AE/FSM branch detects deviations in raw heart rate (HR) or pre-processed resting heart rate (RHR) signals. Users can select RHR mode (e.g., "no steps" criterion), temporal resolution, nightly window length, sequence length, and training regime (healthy-only vs mixed-cohort). **Right (imputation):** The generative branch allows the same signal/windowing choices plus configuration of gap characteristics and model hyperparameters. This modular setup supports controlled training and evaluation across diseases, missingness patterns, and architectures to ensure reproducible performance assessment under realistic constraints.

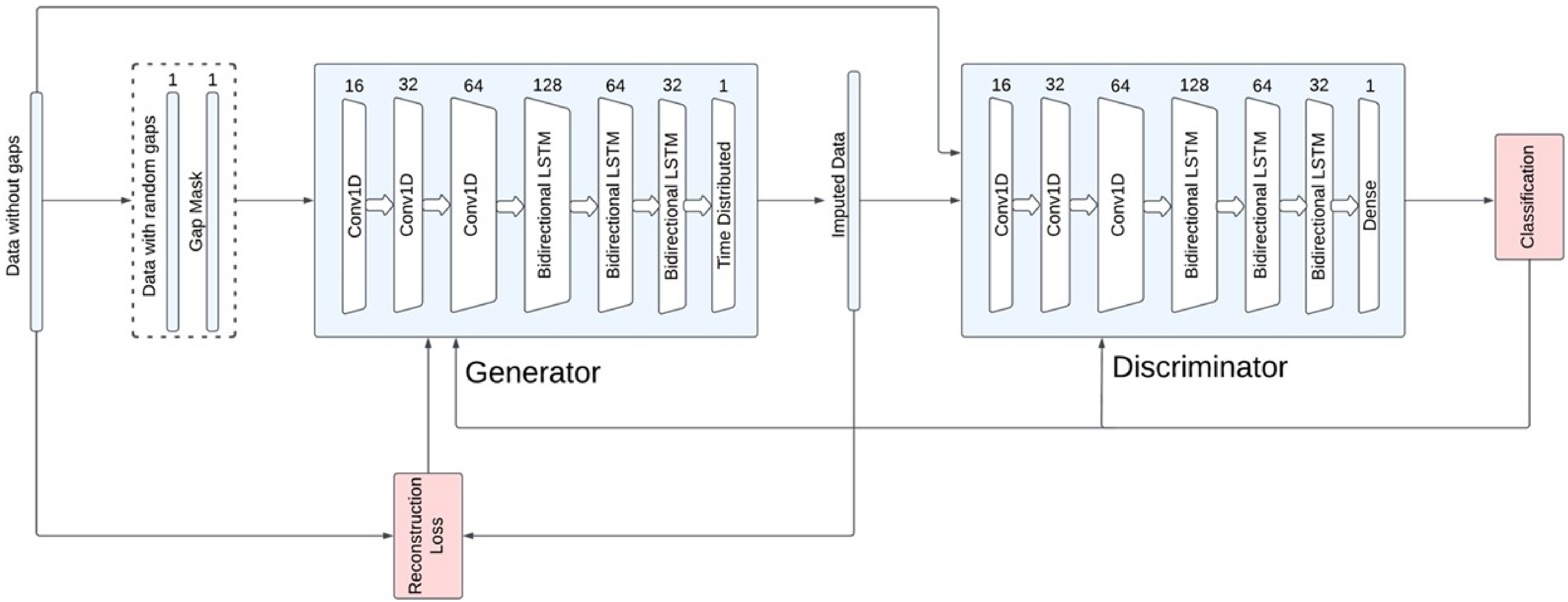

Supplementary Fig. 3: **GAN architecture for heart rate imputation.** A conditional generative adversarial network (GAN) for reconstructing missing segments in wearable-derived heart rate time series. The input sequence without gaps is randomly masked to create artificial missing segments for training, which are passed (together with the gap mask) to the generator. The generator follows a symmetric, U-shaped arrangement of stacked temporal convolutional (Conv1D) and bidirectional LSTM layers, followed by a time-distributed output layer, enabling multiscale reconstruction of the signal. The discriminator, with a similar Conv1D–LSTM architecture, evaluates the generated sequence against real data to enforce temporal coherence and realism. Training is guided by both adversarial loss (from the discriminator) and an L1 reconstruction loss comparing imputed and original sequences. This architecture is optimized for preserving physiological signal structure under varying gap patterns.

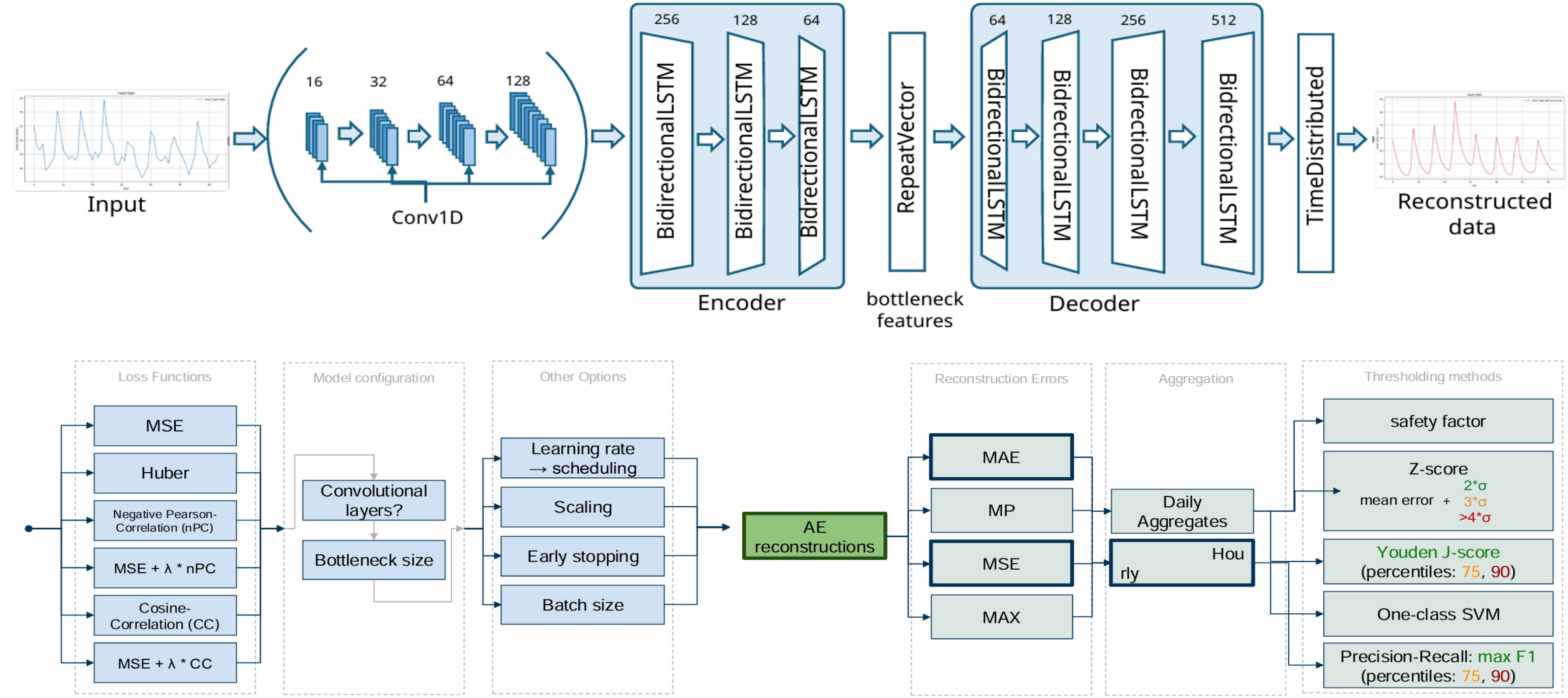

Supplementary Fig. 4: **LSTM autoencoder architecture for anomaly detection.** A composite framework combining model architecture, loss optimization, and post hoc thresholding enables sensitive detection of early physiological deviations. The anomaly detection network follows a symmetric encoder–decoder configuration: stacked Conv1D layers in the encoder extract local temporal features, while bidirectional LSTM layers capture long-range dependencies; the decoder mirrors this structure to reconstruct the signal via a repeat vector and TimeDistributed dense layers, producing per-step error profiles. Input sequences span 7-hour nightly RHR windows over 7 days (49 time steps). To preserve temporal dynamics and physiological relevance, the model was trained using a composite reconstruction loss that combines mean squared error (MSE) with negative Pearson correlation and Huber loss—balancing absolute accuracy with shape fidelity. For anomaly detection, reconstruction errors are evaluated against thresholds defined using multiple strategies, including Z-score cutoffs, ROC curve optimization, Youden's J-index, and one-class SVMs. This modular setup enables benchmarking under realistic noise and missingness conditions, and supports flexible tuning of sensitivity–specificity trade-offs relevant for clinical deployment.

LSTM-AE Model Overview

The architecture for the 49-timestep model is a Keras functional model designed for sequence-based tasks. It processes input sequences of shape (49,1) and produces outputs of shape (49,1). Key components include:

- Input layer with shape (49,1)
- Multiple Conv1D layers for feature extraction
- Bidirectional LSTM layers for sequence processing
- TimeDistributed dense layer for final predictions

1. Input Layer:

- Name: `sequences`
- Shape: (49, 1)

2. Convolutional Layers:

- Conv1D: 16 filters, kernel size 3, `tanh`, same padding
- Conv1D: 32 filters, kernel size 3, `tanh`, same padding
- Conv1D: 64 filters, kernel size 3, `tanh`, same padding
- Conv1D: 128 filters, kernel size 3, `tanh`, same padding

3. Bidirectional LSTM Layers:

- 256 units with L2 regularization ($\lambda = 0.01$)
- 128 units
- 64 units
- 32 units (seq-to-vector)

4. Repeat Vector:

- Repeats vector 49 times

5. Decoder Bidirectional LSTM Layers:

- 32, 64, 128, 256, and 512 units

6. TimeDistributed Dense Layer:

- Dense with 1 unit, `linear` activation

Loss Functions

Mean Squared Error (MSE)

$$\text{MSE} = \frac{1}{n}\sum_{i=1}^{n}(x_i - \hat{x}_i)^2$$

where x_i is the original input, $\hat{x_i}$ is the reconstructed input, and n is the number of data points.

Mean Absolute Error (MAE)

$$\text{MAE} = \frac{1}{n}\sum_{i=1}^{n}|x_i - \hat{x}_i|$$

Huber Loss

$$L_\delta(a) = \begin{cases} \frac{1}{2}(x_i - \hat{x}_i)^2 & \text{if } |x_i - \hat{x}_i| \le \delta \\ \delta(|x_i - \hat{x}_i| - \frac{\delta}{2}) & \text{if } |x_i - \hat{x}_i| > \delta \end{cases}$$

where δ determines the transition between quadratic and linear error penalization.

Negative Pearson Correlation

$$L_{\text{Pearson}} = -\frac{\sum_{i=1}^{n}(x_i - \bar{x})(\hat{x}_i - \bar{\hat{x}})}{\sqrt{\sum_{i=1}^{n}(x_i - \bar{x})^2}\sqrt{\sum_{i=1}^{n}(\hat{x}_i - \bar{\hat{x}})^2}}$$

This loss measures the strength of the linear relationship between the original and reconstructed sequences. By minimizing its negative value, the model encourages correlation between input and reconstruction.

Cosine Similarity Loss

$$L_{\text{Cosine}} = 1 - \frac{\sum_{i=1}^{n} x_i\hat{x}_i}{\sqrt{\sum_{i=1}^{n} x_i^2}\sqrt{\sum_{i=1}^{n} \hat{x}_i^2}}$$

This loss measures the cosine of the angle between two vectors, treating them as direction sensitive signals. Lower values indicate that the reconstruction maintains a similar pattern to the original sequence.

Supplementary Tab. 1: Autoencoder-based anomaly detection model architecture and loss functions.

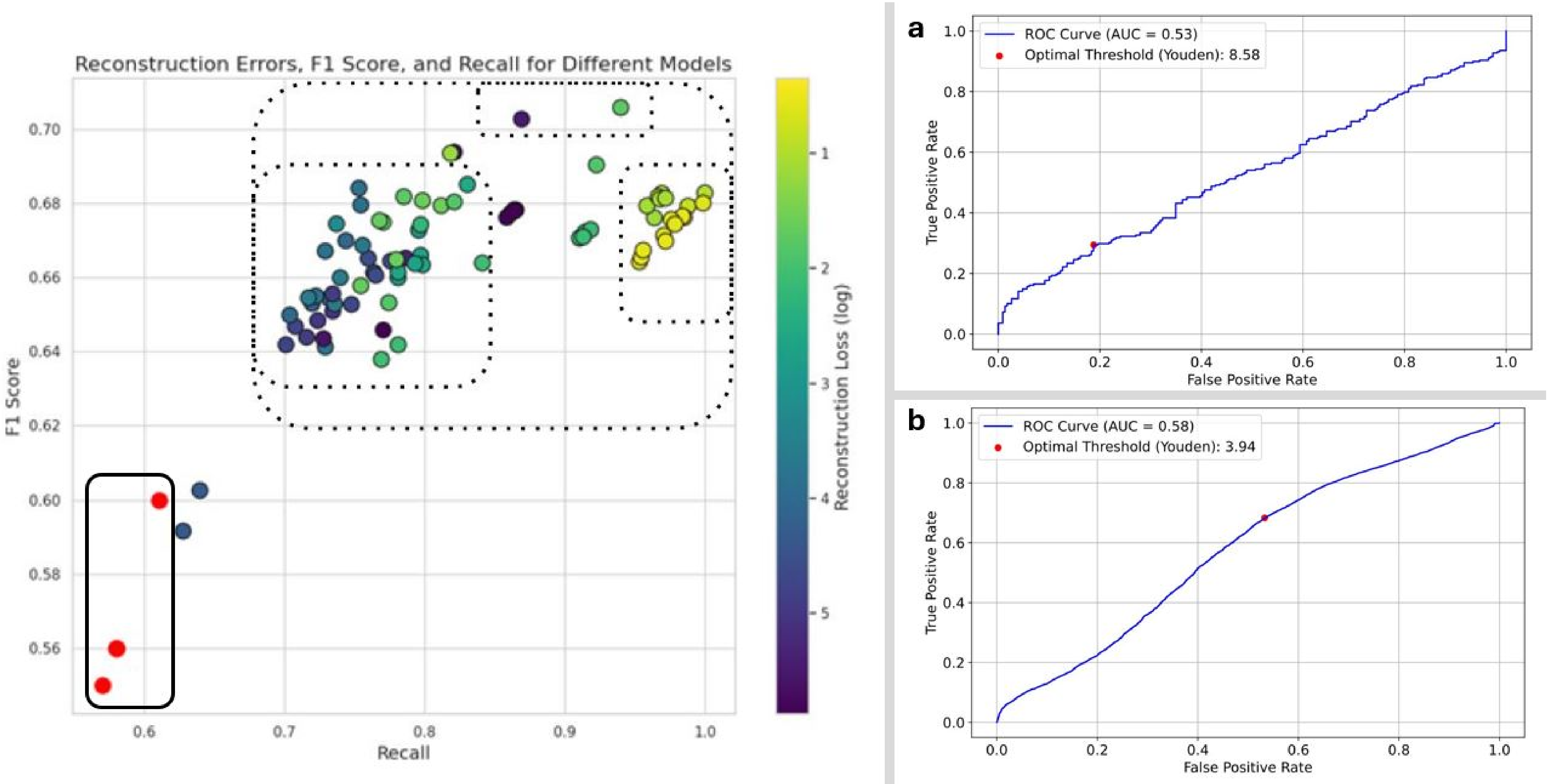

Supplementary Fig. 5: **Comparative evaluation and threshold calibration of the LSTM-AE anomaly detection framework. Left:** Comparison of recall and F1-score across multiple candidate models and LSTM-AE configurations, with point color indicating reconstruction loss (log scale). Models highlighted within the solid-line box correspond to feature-based conventional classifiers and exhibit low recall and F1, demonstrating that reconstruction loss alone is insufficient for reliable case distinction for these approaches. In contrast, LSTM-AE configurations highlighted within the dashed-line box were obtained through grid search and targeted optimization, either minimizing reconstruction error or maximizing F1-score. **Right:** Receiver operating characteristic (ROC) curve for the selected LSTM-AE model based on mean squared reconstruction error for raw (**a**) and GAN-imputed (**b**) sequences. The AUC reflects moderate discriminative performance, consistent with unsupervised reconstruction-based detection. The operating threshold for anomaly detection, determined using Youden's J statistic, balances sensitivity and specificity. Notably, the optimal threshold for GAN-imputed sequences (3.94) is close to the NightSignal threshold (4), suggesting biologically plausible imputation.

RHR Range	Zero Step Window	Original		GAN-imputed	
		TP-rate [%]	FP-rate [%]	TP-rate [%]	FP-rate [%]
0 - 200 bpm	0 minutes	**77.1**	9.56	68.7	7.68
40 - 112 bpm	0 minutes	75.9	9.25	**69.9**	7.48
40 - 102 bpm	0 minutes	**77.1**	8.83	67.5	7.21
0 - 200 bpm	12 minutes	**77.1**	8.95	63.9	7.34
40 - 112 bpm	12 minutes	75.9	8.79	65.1	7.28
40 - 102 bpm	12 minutes	74.7	**8.48**	66.3	**7.16**

Supplementary Fig. 6: **Influence of resting heart rate (RHR) definitions on anomaly detection accuracy in wearable data.** Comparison of how varying definitions of RHR affect the sensitivity and specificity of FSM-based anomaly detection in both original and GAN-imputed signals. The top three rows compare true positive (TP) and false positive (FP) rates across three RHR definitions, where RHR is computed only if the instantaneous step count equals zero in the immediate vicinity of the heart rate measurement. These definitions vary in their temporal strictness, ranging from narrow to broader windows around inactivity. In the bottom three rows, the same RHR definitions are applied with an extended inactivity requirement—enforcing that step count remains zero for a full 12-minute interval surrounding each RHR estimate. This stricter criterion markedly reduces FP rates in both original and GAN-imputed signals, suggesting that short-term physiological fluctuations (e.g., postural changes or recent movement) may confound clinical interpretation. The observations highlight the critical importance of RHR preprocessing strategies in wearable-based disease monitoring and suggest that more conservative definitions improve robustness and reliability of early warning systems.

Supplementary Data

Supplementary Data 1

Malaria study cohort information. Anonymized demographic and study cohort characteristics for all participants included in the malaria analysis. Variables include age range, sex, and malaria detection distribution. These data support cohort description and population-level analyses reported in the manuscript.

Supplementary Data 2

Malaria rapid diagnostic test results. Anonymized rapid diagnostic test (RDT) results for malaria, including test dates and outcomes for participants in the study cohort. These data define infection status and temporal alignment of confirmed malaria episodes with wearable-derived physiological signals.

Supplementary Data 3

Symptom diary records. Longitudinal symptom diary data for participants 279, 294, and 305, including daily reported symptoms and symptom resolution patterns. These data support analyses of symptom progression and recovery in relation to detected physiological anomalies.

Supplementary Data 4

Malaria case detection overview and performance evaluation. Summary tables describing malaria case detection outcomes comparing the rule-based finite-state machine (FSM) approach and GAN-enabled anomaly detection. Included sheets provide an overview of detected cases and pre-symptomatic malaria pattern windows in GAN-imputed cases, as well as performance evaluation metrics for both detection pipelines.
